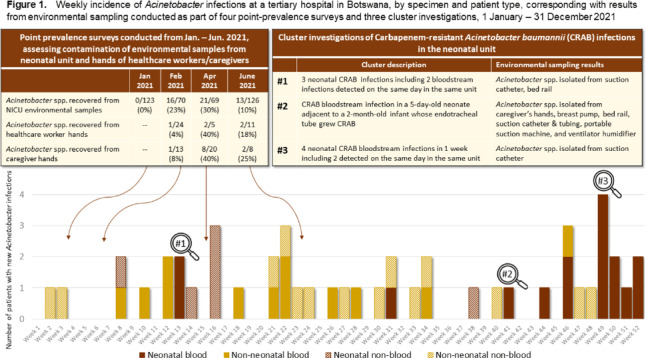# Carbapenem-resistant Acinetobacter baumannii at a tertiary-care hospital in Botswana: Focus on perinatal environmental exposures

**DOI:** 10.1017/ash.2022.206

**Published:** 2022-05-16

## Abstract

**Background:** Bloodstream infections (BSIs) due to carbapenem-resistant *Acinetobacter baumannii* (CRAB) are difficult to treat and are associated with high mortality, particularly in neonates. Healthcare-associated CRAB infections have been linked to environmental reservoirs and are associated with seasonal clustering. CRAB outbreaks are being reported more frequently in sub-Saharan Africa, but published reports from this region that incorporate comprehensive surveillance data and environmental investigations are rare. **Methods:** We reviewed microbiology surveillance records at a 530-bed, public, tertiary-care hospital in Botswana from January 1 to December 13, 2021, and we collected data regarding age, specimen type, and onset date for all cultures from unique patients growing *Acinetobacter* spp. An automated blood-culture system was used for organism detection, manual biochemical tests were used for identification, and disc and agar diffusion methods were used for antimicrobial sensitivity testing. During this time, we conducted 4 point-prevalence environmental sampling surveys at this hospital’s 36-bed neonatal unit from January through June 2021 in addition to 3 neonatal CRAB cluster investigations. Environmental samples from surfaces, hands of caregivers and healthcare workers, and equipment were collected using flocked swabs. Extended-spectrum β-lactamase–producing organisms from environmental samples were identified using selective and differential chromogenic media (CHROMagarTM ESBL). **Results:** Overall, 48 *Acinetobacter* infections were identified, including 28 BSIs (among 3,699 blood cultures processed, approximately one-third of which were from neonates). More than half of cases were perinatal, which included 16 neonatal BSIs (median age, 4 days; case fatality rate, 56%), and 1 fatal case of postpartum sepsis in a 37-year-old mother. Among isolates tested, 35 (92%) of 38 demonstrated carbapenem resistance. Treatment information was not available for all neonatal patients, but delays in appropriate antimicrobial therapy were cited in all fatal cases. Most neonatal CRAB cases clustered in time and space (Fig. 1). For example, 15 (71%) of 21 neonatal cases occurred in the same unit and same week as another case. In the neonatal unit, CRAB clusters were associated with increased *Acinetobacter* recovery during environmental point-prevalence surveys (Fig. 1). *Acinetobacter* contamination was identified on feeding equipment (breast pumps, feeding tubes), respiratory equipment (suction machines or catheters, ventilator humidifiers), and hands of caregivers and healthcare workers. **Conclusions:** We report hyperendemic rates of CRAB infections with evidence of spatotemporal clustering, especially among neonates. Higher CRAB incidence coincided with increased *Acinetobacter* recovery during environmental sampling. We identified plausible transmission vehicles (respiratory or feeding devices, hands) in the neonatal care environment highlighting the value of environmental sampling to support CRAB investigations and reinforcing the importance of comprehensive and consistent disinfection practices, especially in resource-limited settings where equipment is shared or reused.

**Funding:** None

**Disclosures:** None

**Figure:**